# Computational Analysis of a Marine-Derived Drug From Rhizophora mucronata Against the Capsid Protein of Rubella Virus

**DOI:** 10.7759/cureus.67352

**Published:** 2024-08-20

**Authors:** M Protim, KLG Afeeza, S Vasugi, E Dilipan

**Affiliations:** 1 Physiology, Saveetha Dental College and Hospitals, Saveetha Institute of Medical and Technical Sciences, Saveetha University, Chennai, IND

**Keywords:** computational biology, capsid virus, rhizoporin, marine drug, rubella virus

## Abstract

Background

Rubella, commonly known as German measles, is caused by a single-stranded RNA genome. Vaccination is currently the most effective method for preventing rubella and its complications. Molecular docking, a computer-based technique used in drug discovery and development, is used to investigate the interactions between potential drug candidates and their target proteins. It predicts the binding interactions between small molecules (ligands) and the target protein. In this study, we examined a marine-derived drug from *Rhizophora mucronata* for its potential antiviral properties against the rubella capsid virus. Our objective was to identify the active inhibitory sites of the capsid virus.

Materials and methods

Protein and ligand molecules were retrieved from Protein Data Bank (PDB) and PubChem databases. The Lamarckian genetic algorithm was used to calculate molecular docking using Autodock Tools 1.5.7. The docking parameters used for each docked molecule were determined from 100 separate docking experiments with a maximum of 2.5×10^-6^ energy and a mutation rate of 2.0 and mass over ratio of 0.8. The results were recorded as docking parameter files (DPF). PyMOL was used to view and investigate the interactions between ligand fragments and rubella capsid protein.

Results

This approach plays a crucial role in the development of structure-based drugs. The results of the molecular docking suggest that Rhizophorin has the potential to bind with the rubella capsid protein. The strong binding affinity of -6.05 kcal/mol between the ligand and the protein further supports the potential of Rhizophorin as a therapeutic agent. The formation of hydrogen bonds between the ligand and amino acid residues Glu79, Arg82, and Thr118 indicates the significance of electrostatic interactions in the binding process. Furthermore, the hydrophobic interactions between the ligand and residues Ala81, Val84, Leu87, and Ile119 suggest the role of non-polar interactions in stabilizing the complex. The identified amino acid residues involved in these binding interactions could serve as potential targets for drug development. In future studies, experimental validation of the predicted interactions could provide further insights into the potential of Rhizophorin as an antiviral agent.

Conclusion

According to the findings of this study, the in silico investigation successfully identified a target for inhibiting the rubella virus (RuV) capsid receptor molecule. Future investigations on these compounds will require in vitro and in vivo studies using models that are more relevant to the medicinal potential of the capsid protein molecule.

## Introduction

Rubella, a contagious disease characterized by fever and a distinct rash caused by the rubella virus (RuV), poses a significant risk, particularly during early pregnancy, as it can result in the development of congenital rubella syndrome, a condition with serious medical and public health implications. Each year, the World Health Organization reports a substantial number of cases of congenital rubella syndrome, with an estimated 100,000 instances [1]. Maternal rubella infection, especially in the early stages of pregnancy, can have severe consequences, including premature labor, intrauterine growth retardation, congenital rubella syndrome, miscarriage, and, in the most severe cases, intrauterine fetal death [1]. The classic triad of congenital rubella syndrome typically consists of cataracts, congenital heart abnormalities, and sensorineural deafness, with manifestations usually occurring within the first 11 weeks of pregnancy [2]. Since 1969, highly effective vaccinations have been developed, and immunization efforts have been initiated in numerous countries. Despite these advancements, the diagnosis and prevention of rubella still pose challenges [3].

In the realm of eukaryotes, the mechanism of RNA interference (RNAi) plays a vital role as an antiviral defense mechanism. Viruses, in their continuous struggle for survival, often employ viral suppressors of RNAi (VSRs) to overcome this antiviral RNAi response. However, the understanding of how the rubella virus (RuV) counteracts RNAi has long remained unclear [4]. The same study demonstrates that RuV encodes a VSR to evade the antiviral RNAi response, thereby expanding our understanding of the relationship between RuV and its host and uncovering a potential therapeutic target for RuV.

Shifting our focus to the coastal regions of the Indian Ocean, we come across the resilient mangrove trees belonging to the *Rhizophora mucronata* species. These coastal inhabitants, specifically the *Rhizophora *species, have garnered the interest of researchers due to their promising antiviral properties [5]. The presence of flavonoids, particularly catechin, suggests that these mangroves may have inhibitory effects on cholinesterase and also act as antioxidants [6]. A previous study revealed that *Rhizophora mucronata* contains flavonoids, as well as gallic acid, quercetin, and coumarin [7]. Mangroves contain secondary metabolites with potential anticancer properties, particularly antioxidants. These antioxidants can be used as pharmaceutical agents and play a crucial role in maintaining immune system balance [8]. Analysis of mangrove samples has identified seven compounds with antitumor properties. Among them, *Rhizophora* extracts have been found to have high concentrations of 4-pyrrolidinyl, ketone, and pyrazole derivatives, which act as antitumor and anti-inflammatory agents [9]. Furthermore, the bark of *Rhizophora mucronata* and the leaves of *Rhizophora apiculata* contain active polysaccharides that inhibit the binding mechanism of human immunodeficiency virus (HIV) within cells. This inhibitory effect is attributed to the electrostatic interaction facilitated by the negative charge of the acid polysaccharides (sulfate polysaccharides) [10].

In a broader context, the intricate interactions between viruses and their hosts remain a central focus in the ongoing battle against infectious diseases. Both the strategies employed by the rubella virus to counter host defenses and the potential therapeutic applications of naturally occurring compounds found in mangroves serve as examples of the multidisciplinary approach required to address complex health challenges. This comprehensive investigation underscores the necessity for continuous research and collaboration to uncover new insights and solutions in the fields of virology and natural medicine. Thus, the present study aims to analyze the marine-derived drug from *Rhizophora mucronata* against the rubella capsid virus and identify the active inhibitory sites of the capsid virus.

## Materials and methods

Ligand selection

**Figure 1:**
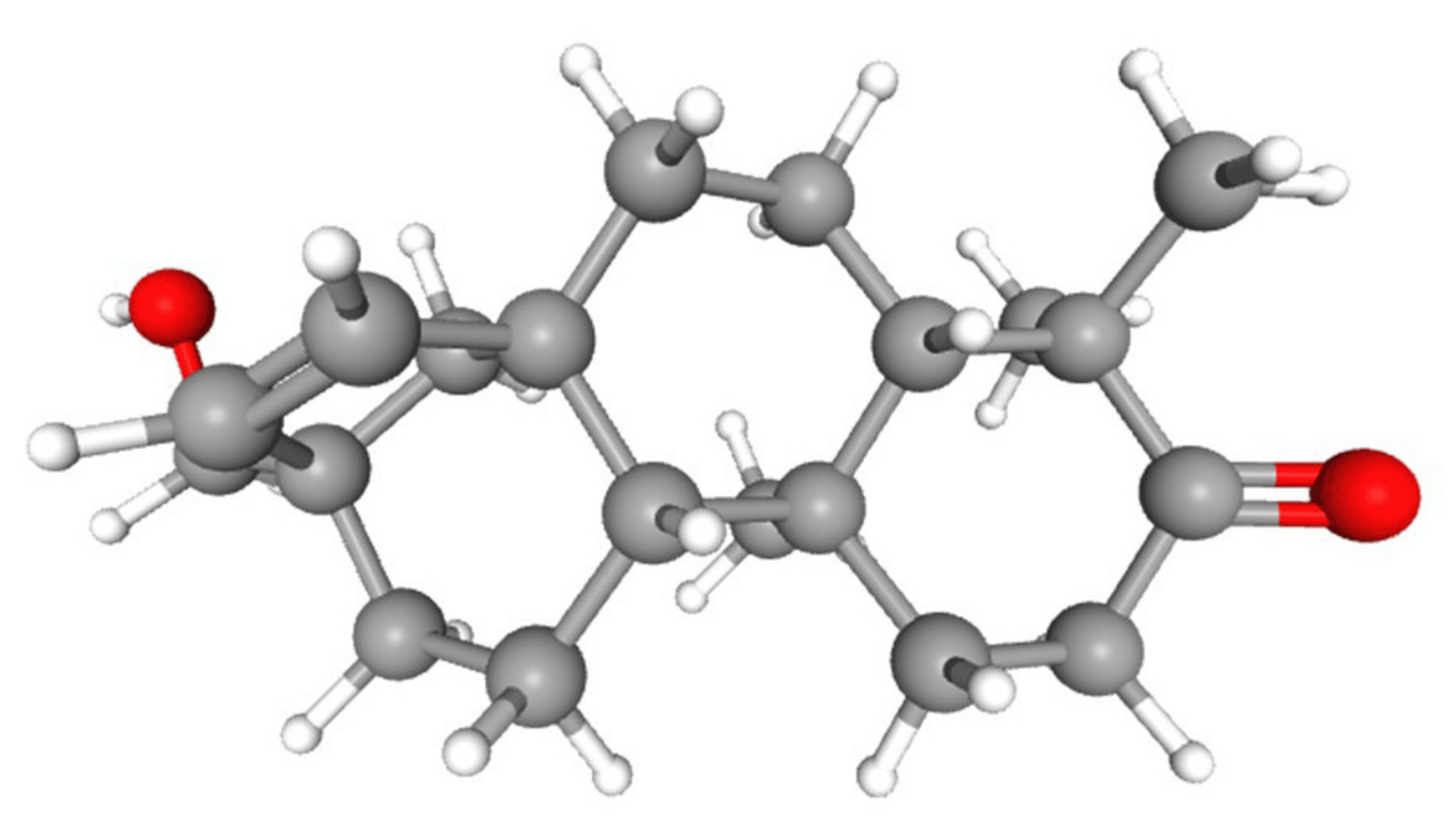
Structure of the ligand (Rhizophorin) molecule retrieved from the PubChem database Name: Rhizophorin, PubChem ID: 42607696, molecular formula: C_25_H_28_O_12_

A search was conducted in the PubChem database to extract the three-dimensional (3D) structures of the Rhizophorin molecule (PubChem ID: 42607696) in structure data file (SDF) format (Figure 1). Subsequently, the 3D structure of the ligands was converted into Protein Data Bank (PDB) format using the OpenBabel software [11]. The AutoDock Tools 1.5.7 was used to set the number of torsions (1-6), apply aromaticity requirements, and set the angle cutoff (7.5).

Protein retrieval and processing

**Figure 2:**
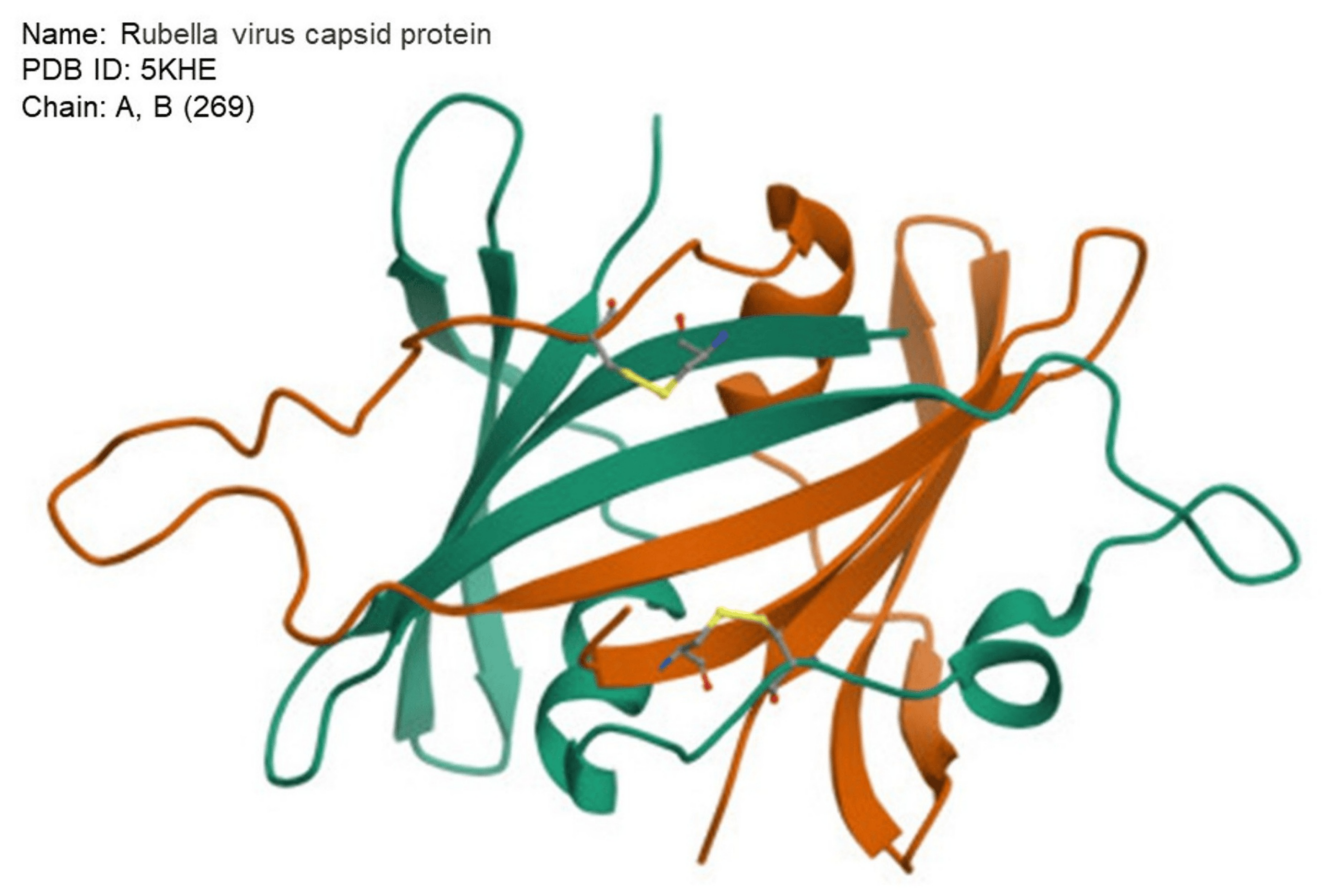
Structure of the protein molecule retrieved from the PDB database

The three-dimensional structures of the rubella virus capsid protein (PDB ID: 5KHE) were obtained from the Protein Data Bank (PDB) database and are depicted in Figure 2. The Discovery Studio Visualizer, a visualization tool, was used to remove excess water molecules and chains from the protein. Prior to docking with a ligand, the receptor molecule underwent preprocessing, which involved removing water molecules and heteroatoms relevant to the docking process. Additionally, any missing atoms in amino acid residues were deleted. Furthermore, the ligand-bound active site in the PDB file data was eliminated using AutoDock Tools [12].

Molecular docking

The molecular docking process was carried out using the Lamarckian genetic algorithm with the AutoDock Tools 1.5.7 software [13]. To create the grid box, AutoGrid was used with dimensions of 50×64×78 Å and a grid spacing of 0.5 Å. The grid box was centered specifically on the hotspot residues within the active site of the target 5KHE. Its coordinates were set at 83.35, 49.60, and 50.60 in the x, y, and z dimensions, respectively. Following the methodology established by White et al. [14], the docking parameters for each molecule were determined based on 100 separate docking experiments. These experiments included a maximum of 2.5×10^-6^ energy assessments, a mutation rate of 0.02, and a crossover rate of 0.8. Docking parameter files (DPF) were used to document the results obtained through the Lamarckian genetic approach. Clustering analysis, with a tolerance of 1.0 root mean square deviation (RMSD), was employed to analyze the predicted binding poses of each molecule. The representative conformation with the lowest energy from the largest cluster was selected. Furthermore, PyMOL, a user-friendly molecular graphics visualization tool for structural bioinformatics analysis [13], was used to observe and analyze the interactions between the ligand fragments and the capsid protein domain of the targets.

## Results

The structure of proteins was analyzed using PyMOL. The ranking of docking poses was determined by the combination of docked ligands, the binding poses corresponding to those ligands, and the docking scores associated with each of those ligands. A grid spacing of 0.375 Å and grid parameters were used in the 10 successful docking runs that were carried out, as described before. Immediately after the simulations, the docked structures were rigorously scrutinized, and the interactions were extensively recorded. To identify the conformers that demonstrated the greatest efficiency, measurements were made to evaluate the hydrogen bond interactions and the binding distance between donors and acceptors. During the investigation, it was discovered that the root mean square deviation (RMSD) tolerance and the van der Waals scaling factor were both set to 1.0 Å for the various conformation clusters.

This approach plays a significant role in advancing structure-based drug development. The results of molecular docking indicate that Rhizophorin has the potential to effectively bind with the rubella capsid protein. The strong binding affinity of -6.05 kcal/mol between the ligand and protein further supports the therapeutic potential of Rhizophorin. The formation of hydrogen bonds between the ligand and amino acid residues Glu79, Arg82, and Thr118 highlights the importance of electrostatic interactions in the binding process (Table 1). Additionally, the hydrophobic interactions between the ligand and residues Ala81, Val84, Leu87, and Ile119 suggest the role of non-polar interactions in stabilizing the complex.

**Table 1:** Molecular docking result of Rhizophorin against rubella virus capsid protein RMSD: root mean square deviation

RMSD	Value
Binding energy	-6.05 kcal/mol
Ligand efficiency	-0.27 kcal/mol
Inhibitory constant	36.73 mM
Intermolecular energy	-6.65 kcal/mol
Total energy	-1.74 kcal/mol
Amino acid residue	Chain A: ALA102; LEU117; ARG118; MET119 Chain B: ARG81; ASN84

**Figure 3:**
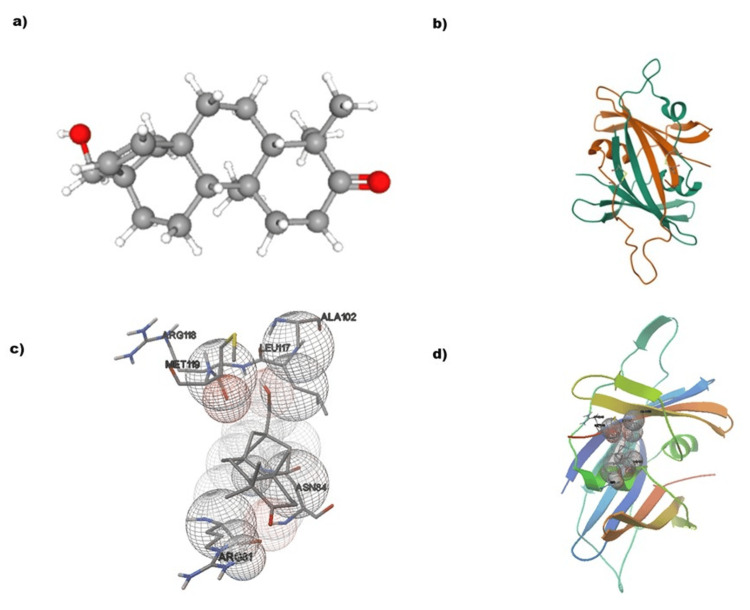
Molecular docking of rubella virus capsid protein with Rhizophorin The receptor interaction with the ligand is represented by a blue color. a: Rhizophorin, b: rubella virus capsid protein, c: protein-ligand interaction, d: ligand molecule docking with A chain of rubella virus capsid protein

Molecular docking is a computational technique used to predict how a small molecule, called a ligand, will bind to a protein receptor. In Figure 3, we can see the docking results of Rhizophorin with the rubella virus capsid protein. The interaction between the two involves specific amino acid residues (ARG118, MET119, LEU117, ALA102, and ASN84) that are labeled. The ligand, Rhizophorin, is represented by spheres, and the blue-colored regions on the protein indicate the binding sites. The gray mesh surrounding the ligand represents the spatial orientation and binding pocket within the protein. The docking pose of Rhizophorin within the A chain of the rubella virus capsid protein is shown. The protein structure is displayed in a ribbon format, with different colored elements highlighting the secondary structure. The ligand, Rhizophorin, is docked in the binding pocket of the A chain, indicating the preferred orientation and molecular-level interactions. The interactions between Rhizophorin and the amino acid residues (ARG118, MET119, LEU117, ALA102, and ASN84) highlight the important contact points. These residues could significantly contribute to stabilizing the ligand within the binding site and, therefore, affect the overall binding affinity.

## Discussion

This research study aimed to evaluate the effectiveness of Rhizophorin, a pharmaceutical compound derived from *Rhizophora mucronata*, against the rubella virus capsid protein. The analysis involved molecular docking to study the interaction between Rhizophorin and the capsid protein, as well as the binding energy of the protein-ligand interaction. The findings showed significant interactions between Rhizophorin and the capsid protein, suggesting potential antiviral activity.

*Rhizophora*, a genus of mangrove trees, has attracted considerable attention due to its potential antiviral properties, particularly its ability to combat the Zika virus [15]. The Zika virus is a pathogen transmitted by mosquitoes, and it is known for its devastating impact on pregnant women and unborn children. Studies have shown that extracts derived from *Rhizophora *species demonstrate antiviral activity against Zika, suggesting that the bioactive compounds present in these plants may have potential in the development of antiviral treatments [16]. Nevertheless, further research is required to fully comprehend the underlying mechanisms and confirm their efficacy. Nonetheless, the discovery of the antiviral potential of *Rhizophora* presents new possibilities for addressing infectious diseases and underscores the importance of exploring natural remedies in the search for effective treatments [17]. The technique known as molecular docking is used to predict how a ligand and a protein interact, resulting in the formation of a stable complex [18]. The goal of molecular docking is to find the best position for the ligand within the protein's binding site and estimate how well they bind together [19]. Figure 3 shows the docking results of Rhizophorin, a naturally occurring compound known for its bioactive properties, with the capsid protein from the rubella virus [20]. Figure 3 uses gray color to represent carbon atoms, red for oxygen atoms, and white for hydrogen atoms.

Previous research studies have underscored the promising prospect of marine-derived compounds as antiviral agents. For instance, investigations have elucidated the antiviral attributes of diverse marine organisms, shedding light on the distinct structural attributes that contribute to their efficacy against a range of viruses [21]. Our findings are consistent with these observations, as they provide evidence that Rhizophorin displays notable binding affinity to the capsid protein of the rubella virus, thus substantiating the potential role of marine-derived compounds in antiviral therapeutic interventions. The structure of Rhizophorin is important because it interacts with the viral capsid protein and has the potential to inhibit it. Figure 3 also shows the three-dimensional structure of the rubella virus capsid protein, with different colors used to represent alpha-helices, beta-sheets, and loops. The capsid protein is essential for the virus to protect its RNA. Inhibitors such as Rhizophorin can target this protein and disrupt the virus's life cycle [22]. Figure 3 highlights the specific interactions between the rubella virus capsid protein and Rhizophorin, labeling the amino acid residues that play a significant role in the interaction. The blue patches indicate the contact sites on the protein, showing where they bind together [23]. It also shows that Rhizophorin fits well into the binding pocket of the capsid protein. This suggests that Rhizophorin could potentially hinder the activity of the capsid protein and limit the virus's ability to infect host cells. The key contact regions between Rhizophorin and the amino acid residues are highlighted, as these interactions likely contribute to the overall binding affinity [24].

Rhizophorin is a good match for the binding pocket of the capsid protein, making it an excellent candidate for binding and potentially suppressing the rubella virus. The present study's molecular docking analysis provides further support for previous research by elucidating the successful interaction between viral proteins and chemicals derived from marine sources. A noteworthy instance of this phenomenon is the investigation conducted by Ouassaf et al. [24], wherein the high binding affinities of marine algae compounds to the HIV-1 protease were demonstrated. This finding indicates the potential of these compounds to function as inhibitors for HIV-1. Our docking data indicate that Rhizophorin exhibits a favorable interaction with the rubella virus capsid protein, as evidenced by its promising binding energies and interaction patterns. The molecular docking study demonstrates that Rhizophorin interacts precisely and efficiently with the binding pocket of the rubella virus capsid protein. These interactions involve several important residues, indicating that Rhizophorin has the potential to interfere with the functioning of the capsid protein [25]. The capsid protein of the rubella virus has been identified as a potential target for the development of antiviral medications. The study conducted by Mangala Prasad et al. [26] underscores the significant role played by the capsid protein in both virus assembly and infection. This finding indicates that directing therapeutic efforts toward the capsid protein may offer a promising avenue for treatment. Our computational research lends support to this notion by illustrating that Rhizophorin can successfully bind to the capsid protein, potentially impeding its functionality and interfering with the viral life cycle. Based on these findings, further experimental validation and development of Rhizophorin as a potential antiviral drug against the rubella virus can be explored.

Limitations

There are several limitations associated with computational analysis, although it provides valuable insights and an initial understanding of the interactions between marine-derived pharmaceuticals from *Rhizophora mucronata *and the capsid protein of the rubella virus. These limitations emphasize the importance of supplementing computational research with experimental validation and considering the complex nature of biological systems in the development of new drugs. To verify computational predictions and ensure the efficacy and safety of proposed therapeutic medications, further research is essential, which may involve both in vitro and in vivo studies.

## Conclusions

The conducted molecular docking analysis showcases the potential of Rhizophorin, a compound derived from *Rhizophora mucronata*, as a novel and promising antiviral drug candidate for combating the rubella virus. These findings contribute to the growing body of evidence supporting the utilization of marine-derived compounds in the development of antiviral therapeutics. Moreover, they present avenues for future experimental investigations to validate and augment the therapeutic efficacy of Rhizophorin.
